# The offense characteristics of maternal filicides in eSwatini: adding to Resnick’s classification model

**DOI:** 10.3389/fpsyg.2024.1456514

**Published:** 2024-10-24

**Authors:** Sifiso Shabangu, Melanie Moen

**Affiliations:** Department of Educational Psychology, Stellenbosch University, Stellenbosch, South Africa

**Keywords:** eSwatini, maternal filicide, Resnick model, socio-cultural motivation, retrospective study

## Abstract

**Introduction:**

Maternal filicide, the murder of a child by a mother, is a complex phenomenon. Relatively little is known of filicide on the African continent, especially in eSwatini. This study highlights the complex dynamics at play when these crimes are committed.

**Methods:**

This retrospective study explored offense characteristics of maternal filicide cases in eSwatini from 2005 to January 2024. Thirty-one cases were identified through court documents and news reports. Content analysis was conducted on these cases.

**Results:**

The study revealed that socio-cultural factors such as the role of a woman in society and traditional beliefs contributed to these crimes. Poverty and contextual challenges also contributed to the systemic reasons for these murders. Relationship challenges featured prominently. A unique finding in this study was the use of poison by biological mothers in all the filicide-suicide attempts.

**Discussion:**

We argue for the inclusion of an additional socio-cultural category to Resnick’s classification model. Ultimately, there is a need for further exploration of filicide in eSwatini and other contexts. This would aid in identifying risk factors in pregnant mothers, in young mothers, among couples, and within socio-cultural practices.

## Introduction

Mothers and children are considered the most vulnerable population in society. The vulnerability of especially children in the home setting is amplified when one considers maternal filicide. Statistics indicate that the home is the most dangerous environment for a child (Moen and Bezuidenhout, 2023). In 2023 the World Health Organization [WHO] (2023) recorded 41, 000 murders in Africa against children under the age of 15 years. Included in these statistics is maternal filicide - when a mother murders her child(ren) (Milia and Noonan, 2022).

Debowska et al. (2015, p. 116) reported that biological mothers, “posed the greatest risk of fatal harm” to their children. Filicidal women often face challenges such as unemployment, financial struggles, and psycho-social problems including exposure to adverse childhood experiences, abusive partners and a history of filicide and suicide attempts (De Bortoli et al., 2013; Lysell et al., 2013; Raymond et al., 2021). Potentially adding to the risk posed by mothers is that they are the nurturers of the child/ren which allows them time and opportunity to contemplate and execute the murder without interference from the outside world. Eriksson et al. (2016) also indicate that filicidal mothers are likely to report mental health problems. In a sample of 17 hospital admitted filicidal women, over half had mental health disorders such as bipolar-or depressive disorder, schizophrenia, psychotic disorders, or personality disorders (Raymond et al., 2021). Similar outcomes were observed in a South African sample where several of the mothers were psychotic at the time of the murder (Moodley et al., 2019). Mental illness and psychosis, therefore heightens the possibility of filicide.

Maternal filicide definitions are expanded to include the killing of a day-old child (neonaticide) and a child who is younger than a year (infanticide) by a female parent, stepparent or caregiver (Frederique et al., 2023). Children vulnerable to maternal neonaticides (killing of a day-old child) are often in the care of mothers who act alone, rarely with the participation of the father or an accomplice (Friedman, 2023; Naviaux et al., 2020). As indicated earlier, a mother generally has isolated time with the child. Maternal neonaticide perpetrators have been found to be young mothers lacking resources (Ben-Nun, 2017), but without a criminal or psychiatric history (Beyer et al., 2008). Young mothers often live with their parents and deny or conceal pregnancies due to fear of consequences (Friedman, 2023), such a losing their economic and social support network (Oberman, 2003). Unmarried women often conceal their pregnancies, because pregnancy is mostly only acceptable within a married relationship. The concealment of pregnancies is associated with lack of prenatal care, which increases the likelihood of filicide tenfold (Frederique et al., 2023). Pregnancy concealment or denial contribute to the lack of attachment and can result in dehumanization of the child which then elevates the risk of neonaticide (McKee and Egan, 2013). Additionally, the mothers who consider neonaticide, often deliver their baby in secret, away from health facilities (Frederique et al., 2023) or at home unassisted (Klier et al., 2019). This is also suggestive of increased likelihood of mortality even if the neonaticidal mother decides against the murder. Due to her being isolated during delivery, far from health and human resources, and being too weak to seek help. Furthermore, women who perpetrate filicides involving older children tend to be married, but often lack support and resources, and are first-time or recent mothers under the age of 30 years old (Ben-Nun, 2017).

Neonaticidal mothers report similar stress levels as other maternal filicide perpetrators (Frederique et al., 2023). Mothers who commit maternal infanticide are likely to exhibit similar histories as neonaticidal mothers, considering that there is only a time factor (24 h) that divides these types of filicides. For example, mothers convicted of infanticide in Hong Kong tended to conceal pregnancies, lacked antennal care, and had no record of mental health disorders (Tang and Siu, 2018) similar to neonaticide perpetrators in general (Beyer et al., 2008; Friedman, 2023).

In an effort to contribute towards understanding, prevention and mitigation of filicide, Resnick (2016) suggests a model of five categories for filicide namely: unwanted child, altruistic, acute psychosis, child maltreatment, and spouse revenge. According to this classification model, altruistic filicide, frequently accompanied by suicidal acts, is motivated by a desire to provide relief from suffering, whether real or imagined (Declercq et al., 2018). Acutely psychotic filicide occurs during a psychotic episode and a rational motive is difficult to discern (Karakus et al., 2003). In unwanted-child filicide, the child is no longer desired by the parents due to being viewed as a hindrance or when uncertainties about paternity surface (Adinkrah, 2003; Agazue, 2021; Resnick, 2016). Child maltreatment is the only unintended filicide often due to abuse, while spouse revenge filicide is motivated by wanting to hurt the spouse or partner (Resnick, 2016).

According to Antić (2017) neonaticides can be classified as active or passive. Active neonaticide is when the parent kills the child directly while passive neonaticide is where the act of killing the child is not carried out but chances for survival are minimized due to abandonment. The active or passive commission of the filicide has a bearing on the method and choice of weapon. Neonaticides and infanticides are often committed using drowning, abandonment/neglect, suffocation, and strangulation (Ben-Nun, 2017; Greenwood et al., 2023; McKee and Egan, 2013). Generally, filicide methodology for children older than a year is more violent and aggressive, and these include shooting, being gassed, drowning, battering, poisoning, defenestration, and stabbing (Frederique et al., 2023; Krischer et al., 2007; Resnick, 2016). This is suggestive of age being a factor in choice of methodology, and by extension, weaponry. Weapons often used in these methods are guns, poison, knives, water for drowning, hands and feet, and other objects that inflict violence and aid lethality (McKee and Egan, 2013; Raymond et al., 2021; Resnick, 2016). The mean age of maternal filicide perpetrators varies across contexts from 26 years in Turkey (Eke et al., 2015), 29.8 years in the USA (Shouse, 2013), 25 years in Fiji (Adinkrah, 2001), 32 years in France (Raymond et al., 2021) and to 26.6 years in Rwanda (Muziki et al., 2022).

In this study, maternal filicide will refer to a biological mother, stepmother, ex-girlfriend/wife, or grandmother who attempts to or murders a child/ren. A child refers to a person under the age of 18 years.

### Background context—eSwatini

eSwatini, often referred to as Africa’s last absolute monarch, has 1.2 million citizens, 43% are under the age of 17 years and 12% under the age of five (United Nations International Children's Emergency Fund, 2022, 2023). English and SiSwati are the two official languages, and the population is predominantly ethnic *emaSwati* (85%) (plural for Swati people). Christianity and indigenous belief systems dominate, but other faiths and ethnicities are also represented (Adinkrah, 2023; Goverment of The Kingdom of eSwatini, 2023). The country has four regions; Manzini, the most populous (33%) followed by Hhohho (29%), with Lubombo and Shiselweni regions with a combined population of 38%. The country has multiple challenges such as high national unemployment rates (24%), inequality, and poverty (59%) (United Nations International Children's Emergency Fund, 2023). These factors are indicted as risk factors for homicidal acts by the United Nations Office on Drugs and Crime (2019). Given this, it is plausible that the likelihood for homicidal acts remains present in eSwatini. Manzini is the economic hub of the country, and the economy is largely dependent on agricultural exports like sugarcane and pulp, with small scale farmers of maize and vegetables for domestic supply and family subsistence (Dlamini et al., 2010; Vilane et al., 2012).

The women in eSwatini are born into a patriarchal system that has prescribed gender roles (Curle, 2017; Nyawo, 2020), and patriarchy continues to “nourish abuse and violence” against women (Simelane, 2011, p. 495). These patriarchal doctrines position women as subordinates whose value is associated with male attachment (heterosexual marriage), child rearing, nursing of sick relatives including in-laws, seating with the body of the deceased as cultural custom, and general obedience (Motsa, 2018; Mthembu, 2018; Nyawo, 2020; Simelane, 2011). Women continue to be victims of gender-based violence (GBV) and there is a persistence of femicide and intimate partner violence cases (Adinkrah, 2023). Growing calls from various sectors of society are advocating for GBV to be declared a national disaster (Nxumalo, 2023). The country’s poverty and breakdown of family structures has also normalized situations of mothers raising a child or children without the support of fathers often in one-room apartments (Curle, 2017). There is a 24% unemployment rate, but this doubles for women and youth at 49% (United Nations International Children's Emergency Fund, 2023). This means mothers who are youth encounter increased economic hardships. The children in eSwatini are exposed to sexual (Breiding et al., 2011), psychological and physical violence (Breiding et al., 2013; Motsa and Morojele, 2016), often at the hands of caregivers or parents, fellow pupils, and teachers (UNICEF, 2021; Sibisi et al., 2024). The neonatal mortality rate stands at 20 per 1,000 live births, owing partly to an under-resourced health system (United Nations International Children's Emergency Fund, 2022). Additionally, 93.7% of infants younger than 23 months are at risk of falling victim to physical (77.8%) and psychological violence (66.6%) (Neubourg et al., 2018).

The murder rate in 2019 in eSwatini among children younger than 14 years was 2.9 per 100,000 people, with the general population rate being 18.5 per 100, 000 (World Health Organization, 2022). In World Health Organization (2022), the country registered 5, 655 deaths, 34% (1,910) in Manzini, 29% (1, 641) in Hhohho, and 10.5% (595) were under the age of 19 years (Central Statistical Office, 2023). Causes of death outside of health facilities were not indicated. This exacerbates the lack of filicide-related data in the country. Furthermore, eSwatini has “an absence of an infanticide law” (Rex v Tsabedze, 305 of 2012, 2020, p. 2) and the high court has proposed that parliament “should consider enacting a criminal offense called infanticide” (Rex v Thwala, 106 of 2006, 2008, p. 15) for filicide-related cases as they currently fall under murder. Filicide being governed by murder legislation presents challenges, firstly this potentially contributes to the country’s “huge backlog and operational inefficiencies” as all murder cases are tried in the high court (Ministry of Justice and Constitutional Affairs, 2023). Secondly, the murder charge does not account for the mental health challenges specific to women’s pregnancy such as peripartum depression. Thirdly, this criminal charge then conceals the pervasiveness and severity of the lethal violence perpetrated against children.

According to Mavuso (2023) eSwatini’s Deputy Prime Minister’s (DPM’s) office has a National Plan of Action for Children (NPoAfC) 2023–2027 which shows that 88% of children, under 14 years, are exposed to psychological violence and that 66% of parents approve of physical violence as a form of discipline within the home. The DPM’s office, which is responsible for the welfare of children, plans to establish a national database of violence against children and to assess the characteristics of this violence (Mavuso, 2023). Violence and its fatal consequences against the country’s children remains severely under-researched and therefore not understood. Filicide is not specifically mentioned in the NPoAfC 2023–2027, although filicide is the most extreme and lethal of forms of violence against children.

This study aims to give an overview of maternal filicides in eSwatini by presenting data on women as perpetrators of filicide. The offense characteristics of these crimes will also be discussed and outlined. This is an under-researched area in general, but specifically in eSwatini with women as perpetrators. With this study we hope to contribute to the knowledge base on filicide, especially on the African continent.

## Methodology

This retrospective study utilized newspaper articles and court cases as sources of data. The Times of Swaziland, established in 1968, boosting a readership of 300,000 in a population of 1.2 million, it is the country’s largest daily newspaper (Times of Swaziland, 2023). The second biggest newspaper is eSwatini Observer, established in 1981 and printed daily (eSwatini Observer, 2024). Both newspapers were accessed via their electronic archives only. The use of newspapers as sources of data is not unprecedented in research in general (Adinkrah, 2023; Cavlak et al., 2023; Jaung and Carrasco, 2022) and, specifically for filicide (Campus, 2016; Moen and Bezuidenhout, 2023; Rizzo et al., 2023; Yoon et al., 2022). Additionally, newspaper reports are acceptable data sources particularly in contexts where official statistics on filicide are often lacking due to absence of filicide-specific data in institutions like the police service (Adinkrah, 2023). Newspaper archives were accessed on their websites https://times.co.sz/archive and https://new.observer.org.sz/. The search period in the newspaper archives was limited to cases beginning in 2009 as the database was not available pre-2009. We began collecting data early February 2024 therefore, included newspaper cases are from 2009 to January 2024. The keywords used in newspaper article retrieval included: ‘killing,’ ‘killed,’ ‘murder,’ ‘murdered,’ ‘filicide,’ ‘mother kills child,’ ‘father kills child,’ ‘suicide,’ ‘murder-suicide,’ and ‘child killed.’ The newspaper databases do not need Boolean operators, namely, AND, OR, and NOT (Aliyu, 2017).

In eSwatini murder cases are tried at the High Court. The court cases were accessed on the Swazi Legal Information Institute (SWAZILII) website https://eswatinilii.org/. This is an open access website of the Judiciary of eSwatini premised on citizens’ rights to information about the laws of the land. The SWAZILII site was utilized in three ways: firstly, keywords, some with Boolean operators AND and OR, were inserted to retrieve cases, and these include ‘filicide,’ ‘child AND murder,’ ‘mother kills,’ ‘mother kills AND/OR murders,’ ‘attempted murder,’ ‘attempted murder AND child,’ ‘child murder,’ ‘parent murder AND child.’ Secondly, a court case would refer to another precedent case, and the referenced case would also form part of the data. Thirdly, the names of perpetrators and/or victims from the newspaper reports were searched to extract court cases, the presumption being the cases had been presented to the High Court.

In keeping with this study’s definition of maternal filicide, cases that were excluded involved: the perpetrator attempting to murder or murdering their partner, or their partner’s love interest as those would qualify as familicide (Liem et al., 2013). Victims 18 years or older and both parents who participated in the filicide were excluded as our focus was on maternal filicides only. We included cases where the child(ren) was/were the only intended targets of the attempted murder or the murder; where suicidality in the perpetrator was or was not present; and the victim was younger than 18 years. A total of 27 newspaper articles and four high court cases were retrieved. The oldest maternal filicide case in court was dated 2005, and thus the retrospective period begun 2005. The four high court cases echo the backlog with recording and uploading cases in eSwatini. Additionally, some perpetrators commit suicide and therefore their murder case would not be recorded in official court documents. This resulted in 31 cases of maternal filicide from the period 2005 to January 2024. The period of cases was not predetermined but dictated by accessed data, which only became available online from 2005.

### Data analysis

Content analysis was used to explore trends, frequencies, and relationships in large amounts of text data and has three phases: preparation, organizing and reporting (Elo and Kyngäs, 2008). In the preparation phase, the cases were read repeatedly for familiarization of data (Polit and Beck, 2004). The inductive content analysis allowed for the organizing phase of the qualitative data by developing codes and categories such as age and occupation (Elo and Kyngäs, 2008). Reporting on the code and categories enables knowledge development about and comprehension of the phenomenon in its context (Vaismoradi et al., 2013). The content analysis resulted in the following categories: age; victim mortality; relationship to victim; relationship status; occupation of perpetrator; day and location of the filicide; motivation; methods and weapons used; as well as suicidality post filicide. When data was not specified, the notation of ‘undisclosed’ was used.

## Findings

In the following section the socio-demographics of maternal filicide perpetrators in eSwatini will be outlined. Following this, the demographics of the child victims of these filicides will be presented. Lastly, spatial characteristics, motivations and methods for the filicides will be outlined.

### Perpetrators

There was a total of 31 filicide perpetrators, 30 (96.8%) were biological mothers, and one a stepmother. All perpetrators were Black African, *emaSwati* woman (plural for Swati people). Most of the perpetrators, 35.5% (11), were in the 21–25 age group, and the least, 6.5% (2), were in the 36–40 age group. There were no perpetrators over the age of 40 years. The second and third highest number of perpetrators were 19.4% (6) in the 26–30 age group, and 16.1% (5) in the 15–20 age group, respectively. More than half (51.6%) of the perpetrators were under the age of 25. The were three (9.5%) cases in which the ages of the perpetrators were undisclosed. Therefore, it is plausible that perpetrators older than 40 years could have been part of the study population.

Most of the mothers, 29% (nine), were single at the time of the filicide and 19.4% (six) were in a relationship, but not married. Six (19.4%) were married, and one (3.2%) was in a polygamous marriage – she was the first wife. There are two legally recognized marriages in eSwatini, Civil Rites and eSwatini Law and Custom (Ministry of Home Affairs, 2020). The latter permits polygamy. In 16.1% (five) cases, the partner had recently abdicated the relationship. The relationship status of perpetrators was undisclosed in four (12.9%) cases. Occupationally, 38.1% (12) were employed, and the same number (12) were unemployed. The nature of the disclosed occupations of the perpetrators appears to be low-skilled jobs such waitressing, street vending or factory work. One perpetrator survived on her grandmother’s pension. Some of the offender’s partners worked as plumbers, security guards, and sugarcane cutters. The perpetrators’ occupational status was undisclosed in 22.6% (7) cases.

Table 1 indicates socio-demographic information of the maternal filicide perpetrators.

**Table 1:** Socio-demographics of maternal filicide perpetrators in eSwatini, 2005-January 2024.

Characteristics of perpetrators	No.	%
Age group (years)		
15–20	5	16.1
21–25	11	35.5
26–30	6	19.4
31–35	4	12.9
36–40	2	6.5
Undisclosed	3	9.5
Relationship to victim		
Biological mother	30	96.8
Stepmother	1	3.2
Relationship status		
Married	6	19.4
Polygamous marriage	1	3.2
Single	9	29
In relationship	6	19.4
Partner ended relationship	5	16.1
Undisclosed	4	12.9
Occupational status		
Employed	12	38.7
Unemployed	12	38.7
Undisclosed	7	22.6

### Victims

There was a total of 40 child victims, 19 (47.5%) boys, 14 (35%) girls, the gender of 7 (17.5%) were undisclosed. 97.5% of the victims were murdered by their biological mothers. 30 (75%) of the victims were deceased due to filicide, and 10 (25%) victims survived the murder attempt. The average age of victims was 2.7 years. Victims who were less than a day old (neonaticides) comprised 32.5% (13) of the victim population. These were murdered almost immediately or within hours of birth. Six (15%) victims were older than 1 day but younger than 12 months (infanticides), and another 13 (32.5%) victims were older than 12 months but younger than 5 years. The age group of six to 10 years had seven (17.5%) victims and only one victim was in the 11-to-15-year age group. Thirty-two of the 40 victims were younger than the age of five.

The following table, Table 2, shows the demographics of victims.

**Table 2:** Demographics of maternal filicide victims in eSwatini, 2005-January 2024.

Characteristics of victims	No.	%
Age group		
Less than 1 day	13	32.5
2 days – 12 months	6	15
13 months – 5 years	13	32.5
6 years – 10 years	7	17.5
11 years – 15 years	1	2.5
Sex		
Male	19	47.5
Female	14	35
Undisclosed	7	17.5
Mortality		
Deceased	30	75
Survived	10	25

### Spatial characteristics

Domestic environments were the dominant locations for maternal filicides in this study, with the dumping of bodies in pit toilets featuring prominently. A few of the murders occurred near a river, open field, and train tracks.

### Motivations

The most common motivations for maternal filicides in this study appeared to be termination of the relationship, 6 (19.4%) cases; lack of financial resources to support the child or children, 5 (16.1%) cases; the father denying paternity of the child, and if the child was regarded as the source of the mother’s problems, 4 (12.9%) cases each. Infidelity functioned in two ways, if the male partner’s infidelity was discovered, 2 (6.4%) cases, and secondly, if the mother’s infidelity resulted in conception, 3 (9.7%) cases. Both instances were significant motivations for child murder. A child being unwanted in a new relationship was motivation in 2 (6.4%) cases, and a partner being abusive was motivation in 1 (3.2%) case. In one case (3.2%), the mother was physically abusive in that she would assault and strangle the 9-year-old child as form of discipline, ultimately resulting in death. The motivation was undisclosed in three cases usually due to the perpetrator committing suicide (Table 3).

**Table 3:** Motivations for filicides perpetrated in eSwatini, 2005-January 2024.

Motivation	No.	%
Partner terminated relationship	6	19.4
Lack of financial resources to support the child	5	16.1
Father of child denied paternity	4	12.9
Child seen as source of problems	4	12.9
Child conceived from infidelity	3	9.7
Undisclosed	3	9.7
Discovered partner’s infidelity	2	6.4
Child unwanted in new relationship	2	6.4
Physically abusive—discipline	1	3.2
Partner abusive towards mother	1	3.2

### Method used

The filicidal mothers in this study used a combination of methods to murder their children. Passive filicide, which involves dumping and neglecting, strangulation, and poisoning was one of the leading filicide methods (13 cases) in this study. Passive filicide refers to an omission of action that would ensure the child’s survival for instance leaving the child to die (Antić, 2017). Dumping and neglecting the baby was common in neonaticides. This method entailed mostly dumping the child, alive or deceased, in a pit latrine toilet within the domestic setting or neglecting the child in an open field, public toilet, or railway track. If the child was deceased before dumping the body, this was often due to strangulation and/or suffocation. For example, a mother first strangled the child, then suffocated the child with blankets until she died, then dumped the child in the pit latrine toilet. Strangulation was the second most common method involved in 10 cases, mostly neonaticides and infanticides were murdered this way. Strangulation was always carried out by hand, and almost always followed by dumping the child in a pit latrine toilet. For instance, an unmarried 18-year-old mother strangled the child, cut the child’s throat, and then dumped the child in a pit latrine.

The third most involved method was poisoning, present in eight cases. Poisoning was used mostly with victims older than a year – only one case involved a 10-month-old baby. Five cases involved multiple victims. This method had the highest number of murder victims, with 14 victims in total. Lastly, cases involving poisoning, apart from one case of a stepmother, were all murder-suicide. There was one *intended* suicide, the mother planned to kill herself, but she did not attempt suicide. Suicide was *attempted* in five cases, and two mothers *committed* (completed) suicide. Therefore, in seven of the eight cases where poison was used, the mothers either attempted or committed suicide. In the eighth case the stepmother did not attempt suicide. All poison-suicide cases involved biological mothers. The most common poisons used in maternal filicides were the Weevil tablet (Aluminium Phosphide) and Master 900 (Methomex 900 SP), which are pesticides used in farming. One case of paraffin poisoning was also noted.

Suffocation occurred in three cases using a blanket, duct tape and an industrial plastic bag, and in the third case, sellotape. In one case, a 29-year-old mother wrapped the five-day-old girl’s nose, mouth, and ears with sellotape and dumped her in a pit latrine toilet. Drowning in a river was used in two cases. A knife was used in one case to slit the child’s throat. Arson was used in one case, where the married 24-year-old mother soaked herself, her three children (9 months, 4 years, and 6 years), and the household items in petrol before setting everything alight. This followed when her partner’s infidelity was discovered. One case presented with arson-elements. The mother delivered and dumped her living baby into a pit latrine toilet. In the early morning hours, she realized that the child was still alive, she then poured smoldering ash from the previous night’s fire over the child. A 20-year-old mother, after concealing the pregnancy and delivering the baby in secret, buried the newborn alive. The child was buried in a shallow grave, in a nearby forest to the family home. Children who were playing in the morning near the forest heard a child cry, and alerted elders who then dug up the child.

### Comparison of findings with Resnick’s categories for filicide

The cases in this study were categorized according to Resnick’s proposed model of filicide classification. Half of the cases, 16 (52%), were unwanted child filicides. The children were unwanted for multiple reasons such as paternity denial by the father, poverty and lack of finances, or when the child was perceived as a hinderance in the mother’s life. These reasons will be elaborated on in the discussion section. There were 10 (32%) cases of spousal revenge filicide which were motivated by discovery of infidelity, relationship termination, and being with an abusive partner. For example, a married 24-year-old mother discovered her husband’s infidelity and soaked herself, their three children and the household items in petrol and set everything alight. This was done to hurt the husband who was reported to have good relations with his children. Altruistic filicides were observed in four (13%) cases where mothers perceived the active or passive filicide as a loving act as they considered their children to be suffering due to their inability to provide for them. There was only one (3%) case of child maltreatment whereby there was a history of physical abuse of the child who was murdered during an episode of corporal punishment. Acute psychosis filicide was not observed in this study. The researchers only had access to court documents and newspaper reports and the possibility exists that there might have been cases of psychosis not reported on.

In this paper we argue for an additional classification category to Resnick’s model, namely socio-cultural factors. Socio-cultural factors such as fear of *muti* (traditional medicine which can be used to bewitch, poison, or induce other negative outcomes), avoidance of the stigmatizing label of *libuya* (one who has failed in marriage and is an embarrassment to the family) (Nyawo, 2014), the social respectability and economic stability associated with marriage, and the rules around child placement following the infidelity of a wife (*livezandlebe*), a derogatory and stigmatizing term referring to a child considered illegitimate who can never be an heir (Cordial et al., 2010; Lamla, 1985; Oparinde et al., 2017), were also observed in this study.

None of the classification categories of Resnick’s model could account for these unique contextual factors. For instance, in one case, the mother conceived a child during a separation from her husband. Eventually, they reunited, she delivered shortly after the reunification. The husband did not accept the child conceived out of wedlock. The maternal grandmother also did not want to look after the child. To exasperate the situation the lover’s side of the family denied paternity. Out of desperation, the mother left the child in an open field. Socio-cultural practices regarding infidelity of a wife permits the husband to reject the child, divorce the wife and further expel her from their marital home. This outcome would render her *libuya*. Socio-cultural practices are very tolerant and permissive of male infidelity and promiscuity, but extremely retributive of women in the same situation (Nxumalo, 1999). Customary laws dictate that the man who impregnated another man’s wife is liable to a fine, which often results in a lover denying paternity. However, pursuing a fine from a lover also exposes the wife as unfaithful and often results in unfavorable treatment by her in-laws. Exposing infidelity can also result in questioning paternity of the couple’s other children. Traditionally the wife is economically dependent on her husband, and she is not allowed to divorce her husband because once a “Swazi woman is married and smeared with red ochre, she must perform certain functions for her husband for the rest of her life…a husband’s death does not end the wife’s obligations” (Ezer et al., 2007, p. 890). The dissolution of Swazi law and custom marriage in is not impossible but extremely difficult to obtain (Adinkrah, 1990).

## Discussion

This study aimed to develop an overview of maternal filicides in eSwatini by presenting data on women as perpetrators of these crimes. In most of the cases in the present study, the perpetrators grappled with multiple challenges which were often exacerbated by limited or no resources. Friedman (2023) contends that filicidal mothers tend to experience resource limitations combined with several other stressors. In this study, the challenges the mothers faced were often related to relationship stressors. The partner-relational stressors included infidelity, paternity denial, termination of the relationship, unwanted children as well as conceiving children outside of the relationship. It is interesting to note that although socio-economic challenges such as poverty and unemployment were highlighted as important contributors to filicide, relationship stressors were dominant motivators for more than half of the filicide cases in this study.

The relationship motivated murders were mostly due to the termination of the relationship by the male partner. The reasons for the termination of the relationship differed. For example, in one case, the husband to an unemployed 21-year-old mother had impregnated another woman and told her (the mother) to leave their marital home. They had two children, 4 and 2 years old. She murdered the children shortly after her husband ended the relationship. Several stressors could have led to these murders. She might have felt loss and rejection when her husband left her, but the fact that she lost her husband, her home and was unemployed, exacerbated the situation. In eSwatini, the sense of identity and womanhood is strongly tied to one being married and having children to continue the man’s family name (Curle, 2017; Nyawo, 2020). A woman is therefore regarded as a ‘real woman’ and worthy of respect when she is in a married relationship. However, if the woman leaves her marital home for whatever reason, she is deemed an embarrassment and a failure in marriage. That woman would then be called *‘libuya’* meaning the one who failed in marriage and is an embarrassment to her family (Nyawo, 2014). This label is linked to her regardless of the reason for the failed marriage. Another case highlighting relational stressors was where the partner, with whom the stepmother was engaged to be married, terminated the relationship shortly before the filicide. The stressors at play were that the promised marriage was not honored, resulting in the stepmother giving the partner’s five-year-old son a poisonous drink when he came home from school. These examples and others highlight the cultural and relationship factors at play when these crimes are committed.

Unique cultural dynamics combined with relationship stressors were also evident in this study. In one case a 33-year-old mother reported years of abuse at the hands of her polygamous husband who would deny her permission to visit her parental home because he feared that she would come back with *muti* and bewitch the rest of the family. Shortly before she poisoned their children (9, 5 and 3 years old), the couple had an argument.

In 32% (13) of the cases neonaticides were committed, almost immediately after birth. In most cases the baby was unwanted. Some of the reasons involved infidelity where the mother was concerned that the father would find out and leave her. Secondly, a lack of finances to support the child, often due to poverty and unemployment were evident in many cases. A third major reason for neonaticides was when the father left the mother during pregnancy. Often the mother would experience this as rejection of herself and the baby. In some of the cases in the study the fathers would deny paternity which resulted in anger (and later murder) projected on the unborn child. In some of the cases where the father denied paternity, the mother realized that there would be no financial or other support forthcoming resulting in her murdering the baby. Fourth, in a few of the cases the child was unwanted, possibly due to shame and embarrassment within the mother’s family and community, especially when the mothers were young and unmarried. Marriage is a valued patriarchal expectation in eSwatini, and one is shamed for having a child outside this institution. Similarly, neonaticide perpetrating mothers have been found to be generally young, unmarried and without plans for the child (Bourget et al., 2007).

The neonaticidal mothers in this study often concealed pregnancies and delivered in isolation outside of a health facility. In several cases the child was delivered in a toilet or within a domestic setting, often unbeknown to family members. The secrecy, not just about the pregnancy, but the delivery too, helps with achieving the filicidal intent (Frederique et al., 2023; Klier et al., 2019). The concealment of pregnancies also allows mothers to distance themselves from the expected child (McKee and Egan, 2013) with a 10-fold chance of filicide (Frederique et al., 2023). The concealment of pregnancies in this study were often motivated by the fear of losing a relationship for instance when the child was conceived due to infidelity, or where the mother did not want to forgo any social and financial support if the pregnancy is discovered. The concealment of pregnancies, as indicated above, was sometimes also motivated by the avoidance of being shamed by the patriarchal system in eSwatini which devalues women who have had a child before marriage and absolves the male as party to the conception of the child (Motsa, 2018; Mthembu, 2018; Nyawo, 2020; Simelane, 2011). These examples once again highlight the dominant cultural and relationship factors that were at play in these murders.

Eighty percent (32 of the 40) of the victims in this study were below the age of 5 years. This finding echoes findings from other contexts that “the younger the child, the greater the risk” for filicide victimhood (Friedman, 2023, p. 13). Additionally, research indicates that paternal filicide victims tend to be older compared to maternal filicide victims (Bourget et al., 2007). In this study, the cultural and patriarchal belief that looking after a young child is solely a female’s responsibility, most likely contributes to the high child murder rates among children less than 5 years and echoes the stressors mothers in eSwatini are exposed to. Mothers raising a child or children without the support of fathers often in less-than-ideal housing is normalized in eSwatini (Curle, 2017). This normalization is testament to the patriarchal underpinnings of eSwatini’s society where child rearing is a female prerogative, and a man cannot see a newborn until the child is 6 months old (Simelane, 2011). This results in limited attachment opportunities between the father and child and places the responsibility of childrearing solely on the mother. In most cases the mothers are supported by female relatives the first 6 months after birth, again limiting any interaction between fathers and newborn children. For instance, in one case where the pregnancy due to infidelity was concealed, the child was delivered in secret, and then buried alive. The grandfather, an elder in the home to whom everything must be reported so he provides guidance and a way forward, due to cultural practices, could not and did not view the child even after this filicide attempt by the mother. Given that the child was under the age of 6 months it was considered culturally taboo.

The majority of the mothers in this study were in the 21–25 age group. According to Ben-Nun (2017) filicidal mothers are often young, first-time or recent mothers, and below the age of 30 years. Of the 31 perpetrators, only six were over the age of 30. Similarly, it appears in eSwatini, the age of the filicidal mother might be significantly lower, similarly to that of countries such as Turkey, Fiji and Rwanda. Most of these filicidal mothers were unemployed, single or had partners with unskilled, minimum wage occupations. This highlights the lack of financial resources to support the child and often, themselves. It has been established that filicidal mothers are often lacking economic resources such as employment and face financial struggles (De Bortoli et al., 2013; Lysell et al., 2013; Raymond et al., 2021). As mentioned before, in eSwatini this status quo is exacerbated by the high rates of poverty (59%) and unemployment (24%), which doubles for women and youth at 49% (n's Emergency Fund, 2023). This means this age group suffers dual consequences as youth and as women, which can leave young mothers feeling overwhelmed, and unsupported with seemingly persistent despair. In England, similar observations were made of mothers convicted of filicide, who were often emotionally driven and in despair of their situation (McKee and Egan, 2013). In this study, financial despair contributed to mothers wanting to hold on to financial support, at all costs. For instance, a 17-year-old mother delivered her child and put the child who was still alive in a plastic bag and dumped him into a pit latrine. The child was saved by a stranger, and mother and child were reunited. In the subsequent years after the reunification between mother (now 23 years old) and son (he was 6 years old), the mother murdered the son by drowning him. At the time of the murder the mother started a new relationship and she found employment. She did not want the child to become a hinderance to her new lifestyle.

Filicides in this study included active and passive filicides. In passive filicides, the child is dumped or neglected, without the mother actively killing the child but simply omitting or severely diminishing chances of survival (Antić, 2017). The majority of the passive killings were by dumping the child in a pit toilet (a hole dug in the ground), which is common in eSwatini homesteads and in low rent dwellings. In few cases the passive filicides involved dumping and neglecting the child in the forest, on train tracks, or in open fields. It can be postulated that by passively dumping or neglecting the child, the mother might feel she is not committing murder. In a way she absolves herself from murder.

Active filicidal mothers in this study used strangulation in a third of the cases. In all the cases they used their hands as weapons. The use of hands eliminates the need for ‘tools’ or ‘weapons’ and it is effective given the fragility of neonates and infants (Ben-Nun, 2017; Greenwood et al., 2023; McKee and Egan, 2013). Almost half of the victims in this study were under the age of 5 years, which might have made it easier to control and strangle the child.

A contextually important finding in this study was the use pesticides such as Weevil tablet and Master 900 in poison related filicides. Access to these pesticides is relatively easy in eSwatini due to domestic agricultural practices (Dlamini et al., 2010; Vilane et al., 2012). In five of the eight poisoning cases, multiple victims, over the age of 1 year, were killed in filicide-suicides. The stepmother was the only exception. Filicide-suicide acts in this study appeared to have been planned, and not a result of a direct reaction after a trigger event. Studies have found that filicide-suicide is mostly related to psychopathology in the mother (Debowska et al., 2015), but in this study the pathology of mothers was not elicited. However, the possibility of untreated underlying psychopathology following extended periods of despair (McKee and Egan, 2013) cannot be ignored.

## Conclusion

In this study, maternal filicide was a result of resource challenges such as unemployment and poverty, and partner-relational stressors. The relationship stressors included a male partner terminating a relationship. Additionally, socio-cultural factors were also found to motivate filicidal actions. The positioning of women in eSwatini’s patriarchal system was one of the common threads of socio-cultural contributions to filicide in this study context. In this study we argue for the inclusion of an additional socio-cultural category to Resnick’s classification model.

Ultimately, there is a need for further exploration of filicide in eSwatini and other contexts. This would aid in identifying risk factors in pregnant mothers, in young mothers, among couples, and within socio-cultural practices.

## Data Availability

Publicly available datasets were analyzed in this study. This data can be found here: http://www.times.co.sz/ and https://eswatinilii.org/. The eSwatini Observer database: http://new.observer.org.sz/.
